# Insights on Antitumor Activity and Mechanism of Natural Benzophenanthridine Alkaloids

**DOI:** 10.3390/molecules28186588

**Published:** 2023-09-13

**Authors:** Rui Peng, Mengwei Xu, Baocheng Xie, Qing Min, Siwen Hui, Ziwei Du, Yan Liu, Wei Yu, Shi Wang, Xin Chen, Guang Yang, Zhaofang Bai, Xiaohe Xiao, Shuanglin Qin

**Affiliations:** 1 Hubei Engineering Research Center of Traditional Chinese Medicine of South Hubei Province, School of Pharmacy, Xianning Medical College, Hubei University of Science and Technology, Xianning 437100, China; 2 Department of Pharmacy, The Tenth Affiliated Hospital of Southern Medical University (Dongguan People’s Hospital), Dongguan 523059, China; 3 Senior Department of Hepatology, The Fifth Medical Center of PLA General Hospital, Beijing 100039, China; 4 China Military Institute of Chinese Materia, The Fifth Medical Center of PLA General Hospital, Beijing 100039, China; 5 The State Key Laboratory of Medicinal Chemical Biology, College of Pharmacy, Nankai University, Tianjin 300071, China

**Keywords:** benzophenanthridines, natural products, cancer, antitumor activity, anticancer drug

## Abstract

Benzophenanthridine alkaloids are a class of isoquinoline compounds, which are widely found in the plants of *papaveraceae*, *corydalis*, and *rutaceae*. Biological activities and clinical studies have shown that benzophenanthridine alkaloids have inhibitory effects on many cancers. Considering that the anticancer activities and mechanisms of many natural benzophenanthridine alkaloids have been discovered in succession, the purpose of this paper is to review the anticancer effects of benzophenanthridine alkaloids and explore the application potential of these natural products in the development of antitumor drugs. A literature survey was carried out using Scopus, Pubmed, Reaxys, and Google Scholar databases. This review summarizes and analyzes the current status of research on the antitumor activity and antitumor mechanism of natural products of benzophenanthridine from different sources. The research progress of the antitumor activity of natural products of benzophenanthridine from 1983 to 2023 was reviewed. The antitumor activities of 90 natural products of benzophenanthridine and their related analogues were summarized, and the results directly or indirectly showed that natural products of benzophenanthridine had the effects of antidrug-resistant tumor cell lines, antitumor stem cells, and inducing ferroptosis. In conclusion, benzophenanthridine alkaloids have inhibitory effects on a variety of cancers and have the potential to counteract tumor resistance, and they have great application potential in the development of antitumor drugs.

## 1. Introduction

Tumor refers to a disease with the highest mortality rate. Under the action of a wide variety of factors, cells in local tissues lose their normal regulation of growth at the gene level, thus causing abnormal cell proliferation [1]. Over 60% of anticancer drugs have been derived from natural products and natural product derivatives [2].

**Figure 1:**
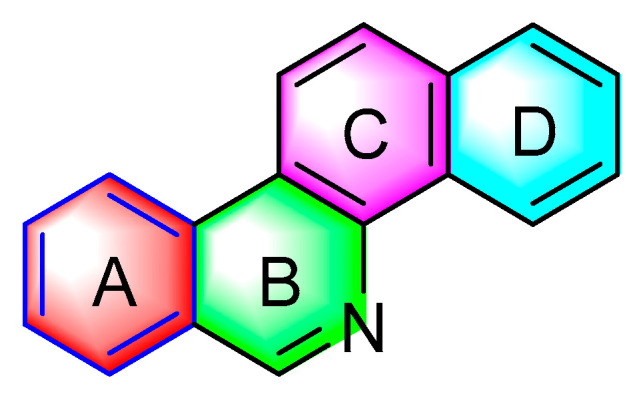
Chemical structure of benzophenanthridine alkaloids.

Figure 1 presents the structure of the benzophenanthridine compound, containing a non-aromatic heterocycle (B ring). It is primarily distributed in *papaveraceae* and *rutaceae* [3]; pertains to isoquinoline alkaloids; and exhibits antitumor, antifungal, antiviral, anti-inflammatory, immune regulation, and other pharmacological activities [4].

Indeed, benzophenanthridine alkaloids play an anticancer role via different mechanisms. Benzophenanthridine alkaloids are capable of affecting the activity of DNA topoisomerase I and topoisomerase II, suppressing the rapid proliferation of tumor cells [5], inducing cancer cells ferroptosis [6], inhibiting the growth of tumor stem cells [7], and so forth. Benzophenanthridine natural products combine with negatively charged membrane surfaces and proteins, and react with sulfhydryl residues of amino acids, thus interfering with collagenase, tubulin assembly, Na^+^/K^+^ATP ase, as well as other functions [8]. The antitumor activities of benzophenanthridine alkaloids are reviewed to lay a theoretical basis for the development of novel antitumor drugs with natural benzophenanthridine alkaloids as the lead compounds.

## 2. Antitumor Activities of Benzophenanthridine Alkaloids from Natural Products

### 2.1. Antitumor Activities of Benzophenanthridine Alkaloids from Papaveraceae Plants

#### 2.1.1. Antitumor Activities of Benzophenanthridine Alkaloids from *Papaver SPP*

**Figure 2:**
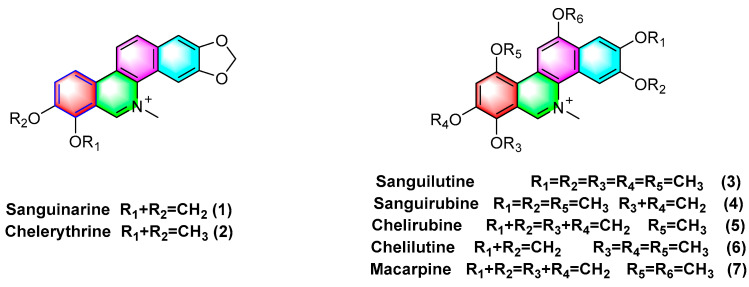
Benzophenanthridine alkaloids from *Papaver* spp.

Quaternary benzophenanthridine alkaloids (QBAs) primarily originate from plants of the genus *Papaver*. Representative compounds comprise sanguinarine (**1**), chelerythrine (**2**), sanguilutine (**3**), sanguirubine (**4**), chelirubine (**5**), chelilutine (**6**), and macarpine (**7**) (Figure 2).

The presence of moderate or high levels of ROS in tumor cells affects the initiation and proliferation of cancer to a certain extent. Benzophenanthridine natural products are capable of inducing, or activating different molecular signal transduction, activating proteins of related pathways, or making them apoptosis by interfering with the signal pathway of ROS, and treating and eliminating tumor cells by regulating dysfunctional proteins [9]. Sanguinarine (**1**), one of the most famous benzophenanthridine alkaloids, can act on ROS-dependent mitochondria to induce autophagy and apoptosis, or inhibit the mitosis of cancer cells by changing the acidic conditions of lysosomes and interfering with the formation of autophagosomes lysosomes, such that liver cancer [10] and MDA-MB-231 human breast cancer [11] can be inhibited. Sanguinarine (**1**) is capable of significantly targeting ephrin type-B receptor 4 (EphB4) and hypoxia inducible factor-1α (HIF-1α) in breast cancer, inhibiting the activation of the downstream protein signal transducer and activator of transcription 3 (STAT3) in cells, blocking hypoxia-induced HIF-1α or STAT3 interacts, and downregulating the mRNA levels of its target genes, thus inhibiting breast cancer cell hyperplasia [12]. In addition, in the breast cancer model, sanguinarine (**1**) has been proven to have the effects of inhibiting the metastasis of breast cancer and anti epithelial mesenchymal transformation (EMT) [13]. A recent review article also showed that sanguinarine (**1**) is a very promising therapeutic option for breast cancer [14]. Sanguinarine (**1**) facilitates apoptosis in HeLa cells as a treatment for cervical cancer with an IC_50_ value of 3.5 μM [15]. Sanguinarine (**1**) exhibits anti-microtubule activity while inhibiting the binding of colchicine and podophyllotoxin to tubulin with IC_50_ values of 32 μM and 46 μM. The IC_50_ values for chelerythrine (**2**) have been obtained as 55 μM and 60 μM [16]. Sanguinarine (**1**) induces apoptosis in HeLa cells by upregulating the expression of the proapoptotic protein Bax and inhibiting the antiapoptotic protein BcI-2, and 0.5 μM sanguinarine treatment leads to a significantly reduced number of colonies formed by HeLa cells [15]. Sanguinarine (**1**) is cytotoxic to different resistant cancer cell lines, and the main mechanisms of action are the inhibition of P-glycoprotein transporters, NF-kB activation, and so forth. For CCRF-CEM, CEM/ADR5000, U87MG, U87ΔEGFR, MDA231, MDA-BCRP, p53^+/+^, p53^−/−^, HEK293, and HEK293/ABCB5 cell lines are significantly inhibited with IC_50_ values of 0.3–4.1 μM [17].

Moreover, sanguinarine (**1**) exhibits strong cytotoxicity against non-small cell lung cancer (NSCLC) with an IC_50_ value of 2.19 μM. Its mechanism of action is likely to be correlated with blocking NF-κB, and Akt and ERK1 signaling pathways have a correlation with the inhibition of cancer cell migration [18]. Sanguinarine (**1**) inhibits the proliferation of BGC-823 gastric cancer cells by downregulating the expression of miR-96-5p and miR-29c-3p and upregulating the expression of MAP4K4, pMEK4, and pJNK1 protein in gastric cancer cells BGC-823 [19]. Sanguinarine (**1**) inhibits human prostate cancer cells by inducing ROS-dependent Par-4 cleavage and increasing ROS concentrations in cancer cells. Moreover, it induces growth arrest and apoptosis of human prostate cancer cells PC3 and DU145 with active caspases, which are activated at 2 μM concentration, such that their colony formation can be inhibited [20]. The existing research has also suggested that long-term treatment with sanguinarine (**1**) causes telomere attrition and cell growth retardation, such that cancer cells become senescent. The main mechanisms are associated with the downregulation of the reverse transcriptase *hTERT* gene expression and inhibition of telomerase activity [21]. Sanguinarine (**1**) induces apoptosis in human HT-29 cells, demonstrating potential therapeutic applications in the treatment of colon cancer [22].

Sanguinarine (**1**) overexpresses inducing long non-coding RNA casc2 in SKOV3 cells or inhibits the NF-kB signaling pathway, thus inhibiting SKOV3 cell growth, proliferation, migration, invasion, and so forth, while ultimately facilitating apoptosis [23]. Moreover, it enhances the sensitivity of cisplatin-resistant cells’ ovarian cancer A2780 to cisplatin [24]. Sanguinarine (**1**) also has potential therapeutic effects against several cell lines with leukemia (e.g., HL-60, drug-resistant HL-60/MX1, drug-resistant HL-60/MX2 (acute promyelocytic leukemia), J45.01 (acute T cell leukemia), U266B1 (myeloma), CCRF/CEM, and CEM/C1 (acute lymphoblastic leukemia)). To be specific, the most potent activity is observed against drug-resistant HL-60/MX2 with IC_50_ values of 0.10 ± 0.05 [25]. Sanguinarine (**1**) can inhibit the migration of 786-O cells in vitro and in vivo, and reverse the epithelial−mesenchymal transition with IC_50_ values of 0.5959 μM [26].

Moreover, recent research has confirmed that sanguinarine (**1**) is capable of inducing H_2_O_2_-dependent cellular ferroptosis in human cervical cancer (Hela) based on a major mechanism that is correlated with the downregulation of SLC7A11 and the depletion of GSH [6]. Besides the above cancer cells, existing studies have found that sanguinarine (**1**), which specifically targets lung cancer stem cells, is a natural anti-lung cancer drug-resistant compound. Furthermore, sanguinarine (**1**) inhibits pancreatic cancer stem cells by inhibiting the sonic hedgehog signaling pathway [27]. The therapeutic effect of sanguinarine on various tumors has been verified at the animal level [28,29,30]. Overall, sanguinarine (**1**) has anticancer potential and is expected to become a leading compound of anticancer natural products [31].

Extensive research has confirmed that chelerythrine (**2**) is capable of affecting estrogen signaling pathways (e.g., the estrogen receptor ER- α 36, ER- α 66, ER-β1, and Src expression), inhibiting gastric cancer cell (AGS) growth proliferation, and facilitating their apoptosis [32]. Chelerythrine (**2**) can act on tgfb1-erk1/2/Smad2/3-snail/ZEB1 signaling to inhibit the progression of cell lines U251 and T98G of glioblastoma (GBM) (e.g., proliferation, migration, stemness, and invasion [33]). In addition, chelerythrine (**2**) also promotes Drp1 mitochondrial translocation to enhance glioma cell lines necroptosis [34].

In human hepatocellular carcinoma (HCC), chelerythrine (**2**) can inhibit human hepatocellular carcinoma Hep3B cells by downregulating the expression of p-FAK and MMP-2/9. Moreover, the main mechanism of action is correlated with the alteration of phosphoinositide 3-kinase (PI3K), Akt, and the mammalian target of rapamycin (mTOR) signaling pathways [35]. Chelerythrine (**2**) has exhibited anticancer activity in vivo and in vitro, and considerable existing research has confirmed that chelerythrine can act on different pathways (e.g., DNA, MAPK, apoptosis, ROS, cell cycle, autophagy, tumor metastasis, and PKC) to inhibit or facilitate apoptosis in a variety of cancer cells (e.g., non-small cell lung cancer [36], prostate cancer [37], lung adenocarcinoma [38], renal cancer [39], and melanoma cells [40], colorectal cancer [41]), thus suggesting that the benzophenanthridine alkaloids exhibit high anticancer activity. Previous studies have proven that chelerythrine (**2**) exhibits antitumor stem cell properties, which are mediated by the downregulation of β- Catenin expression, thus inhibiting non-small cell lung cancer stem cells [42]. Both sanguinarine (**1**) and chelerythrine (**2**) have anticancer activities on human breast cancer cells, but sanguinarine (**1**) has more potential [43]. However, chelerythrine (**2**) has been reported as a promoter that can regulate c-MYC oncogenes, which has become a new strategy to develop anticancer molecules [44].

**Figure 3:**
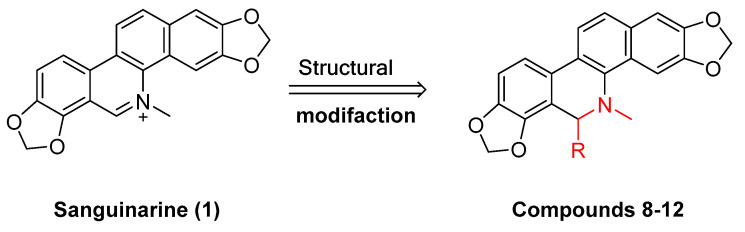
Study on structure–activity relationship of sanguinarine.

**Figure 4:**
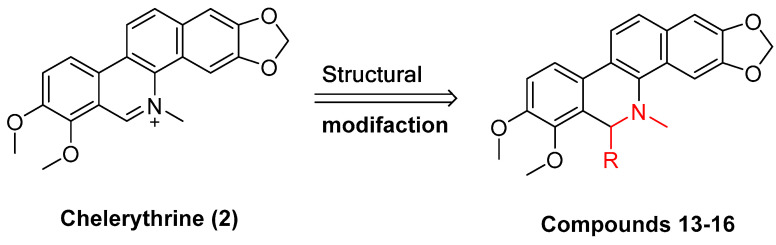
Study on structure–activity relationship of chelerythrine.

Existing research has confirmed that the hydroxymethyl group at the C-6 position of benzophenanthridine alkaloids takes on a critical significance to cellular activity, and the introduction of different groups at the C-6 position can change their activity (Figure 3 and Figure 4) [45]. For instance, the introduction of malonate, dialkylphosphite, and nitroalkanes significantly enhances their cytotoxicity. Furthermore, derivatives (**8**–**12**) (Table 1) obtained after the insertion of the electron-donating group at the C-6 position by sanguinarine exhibit higher cellular activity than those of chelerythrine (**13**–**16**) (Table 2).

The compounds chelerythrine (**2**), structurally modified to yield compounds **13**–**16**, sanguinarine (**1**), and derivatives **8**–**12**, exhibit potent activity against Jurkat clone e6-1 and THP-1 leukemia cell lines, with IC_50_ values from 0.18 to 7.94 μM. Notably, most of the activities of the above compounds are higher than those of chelerythrine (**2**) and sanguinarine (**1**)’s original activities, in which the IC_50_ values of compound **12** against the above two leukemia cell lines reach 0.53 ± 0.05 and 0.18 ± 0.03 μM, respectively [46].

Sanguinarine (**1**), chelerythrine (**2**), sanguilutine (**3**), sanguirubine (**4**), chelirubine (**5**), and macarpine (**7**) (Figure 1) exhibit antitumor activity against various cancer cell lines, including human leukemia (e.g., HL-60, THP-1, MT-4, CEM, and U937), human prostate cancer (e.g., DU-145, LNCaP, and PC3), human epidermoid carcinoma (e.g., A431, nheks,), human pancreatic cancer (e.g., AsPC-1 and BXPC-3), human melanoma (e.g., M4Beu, A372, OCM-1), human non-small cell lung cancer (A549), human breast cancer (e.g., MCF-7 and MDA-MB-231), human ovarian adenocarcinoma (OVCAR-3), cervical cancer (e.g., hen-16-2 and HeLa), human colon cancer (e.g., HCT-116, SW480), and human gastric cancer cells (BGC-823) with IC_50_ values < 10 μM [47].

Sanguinarine (**1**), chelerythrine (**2**), chelirubine (**5**), macarpine (**7**), and sanguirubine (**4**) inhibit HL-60, KF-II, A431, and HeLa activity with IC_50_ values ≤ 0.7 (μg/mL). Of these, macarpine (**7**) shows the optimal activity, with IC_50_ values against four cell lines (μg/mL) followed by 0.012, 0.013, 0.024, and 0.015 [48].

*Sanguinaria canadensis* L. can extract and purify sanguilutine (**3**). Some research has suggested that the antiproliferative activities of sanguilutine (**3**) and chelilutine (**6**) are related to the induction of oxidative stress. As indicated by the results, against three different cancer cell lines, HeLa, A2780, and HL-60, the IC_50_ values of sanguilutine range from 0.04 to 0.46 (μg/mL), and the IC_50_ values of chelilutine (μg/mL) range from 0.16 to 0.84 [49].

Hammerová, Jindřiš Ka et al. [50] further elucidated the mechanism of sanguilutine (**3**) in inducing apoptosis in melanoma cells. As a result, sanguinarine caused a decrease in the mitochondrial membrane potential and levels of antiapoptotic proteins of the bcl-2 protein family, BCL XL, and myeloid cell leukemia protein 1 (Mcl-1), as well as downregulated levels of the X-linked inhibitor of apoptosis protein (XIAP) to facilitate melanoma cell apoptosis.

#### 2.1.2. Antitumor Activities of Benzophenanthridine Alkaloids from *Corydalis saxicola Bunting*

**Figure 5:**
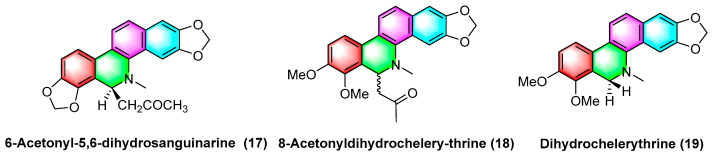
Benzophenanthridine alkaloids from *Corydalis saxicola Bunting*.

The 6-acetyl-5,6-dihydrosanguinarine (**17**), 8-acetyldihydrochelerythrine (**18**), and dihydrochelerythrine (**19**) (Figure 5) are isolated from *Corydalis saxicola Bunting*, and the above alkaloids have some antitumor effects on squamous cell carcinoma, lung cancer, and liver cancer of the tongue. To be specific, the mechanism of action against human tongue squamous cell carcinoma may be inhibiting NF-κB activation, downregulating BcI-2 protein expression on mRNA, and reducing telomerase activity; inhibiting non-small cell lung cancer A549 cell proliferation, migration, and inducing apoptosis; inhibiting proliferation and migration and upregulating the intracellular NF-κB p65 expression of the subunit [51].

**Figure 6:**
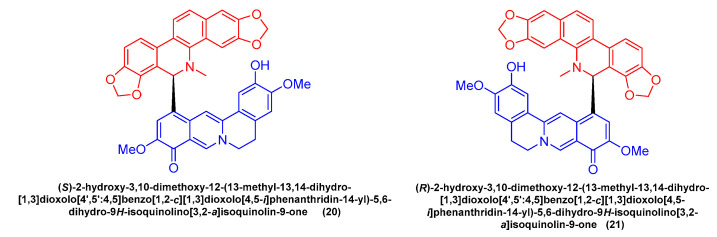
Benzophenanthridine alkaloids from *Corydalis saxicola.*

Feng Qin et al. [52] extracted and isolated two novel dimeric benzophenanthridine alkaloids (**20**, **21**) (Figure 6) from *Corydalis saxicola*, which comprised a mixture of benzophenanthridine and protoberberine passing between the 6, 12 C-C σ Bond direct coupling generation. Compounds **20** and **21** inhibited T24 cells with IC_50_ values of 13.26 μM and 9.45 μM.

#### 2.1.3. Antitumor Activities of Benzophenanthridine Alkaloids from Chelidonium

**Figure 7:**
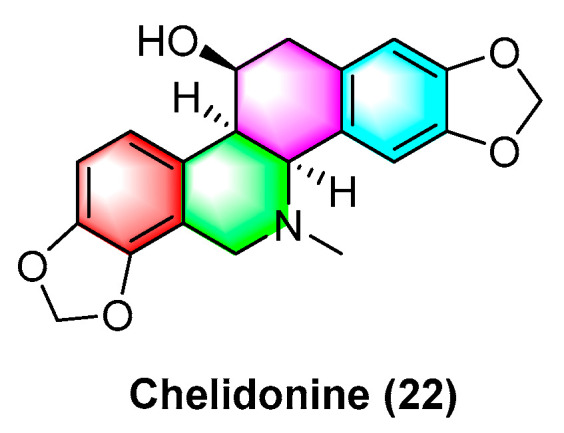
Benzophenanthridine alkaloid from *Chelidonium.*

Sakineh Kazemi noureini et al. [53] have indicated that chelidonine (**22**) (Figure 7) can inhibit MCF-7 in a dose-dependent manner, including cell senescence, apoptosis of autophagic syncytial cells by inhibiting telomerase activity, and chelidonine (**22**) at a 0.05 μM concentration, thus inducing senescence in MCF-7 cells with an LD_50_ value of 8 μM. Chelidonine (**22**) is capable of inhibiting the pharmacological activity of the NRAS activator stk19 kinase. It inhibits arginine, lysine, and leucine (Q61R, Q61K, and Q61L) in NRAS mutant melanoma at glutamate 61 (Q61), thus inhibiting the downstream signaling pathways of RAS proteins (e.g., Raf/MEK and P13K-AKT). As a result, cellular senescence and apoptosis are caused. Chelidonine inhibits STK19 kinase with an IC_50_ value of 123.5 ± 19.3 Nm [54]. Csomós, I., et al. [55] have indicated that chelidonine (**22**) arrests the G2/S phase of melanoma cells by inhibiting the phosphorylation of complexine and serine in the STAT3 signaling pathway in human melanoma cells, thus inhibiting melanoma. The inhibition effect is significant at 1 μg/mL concentration. It has also been documented that chelidonine (**22**) can inhibit growth, invasion, angiogenesis, and suppress gene expression in head and neck cancer cell lines. At 10 μg/mL, it significantly inhibits FADU, HLaC78, HlaC79, and HLaC79-Tax cell lines [56]. Radim havelek et al. [57] indicated that chelidonine (**22**) can inhibit the cell cycle of leukemic T cells in different p53 states while suppressing tubulin polymerization in A549 cells. The IC_50_ value of different tumor suppressor proteins of the *p53* gene for MOLT-4, HL-60, U-937, Raji, Jurkat, and others ranges from 2.2 to 5.0 μM. For non-small cell lung cancer cells (NSCLC), chelidonine (**22**) has strong inhibitory effects, which are achieved primarily through the ability to selectively inhibit the EGFR phosphorylation and inhibit mitochondrial function in EGFR double mutant cells. The IC_50_ of chelidonine after 72 h treatment of various NSCLC cell lines (e.g., H1975, PC9, H460, and H358) ranges from 2.58 to 12.77 μM. Against A549, CCD19 is less active with IC_50_ value > 20 μM [58]. Chelidonine (**22**) can induce cell death in T98G cells through two apoptotic pathways: caspase-dependent and caspase-independent. As a result, cell mitosis is arrested, thus causing cell death, and inhibiting human glioblastoma. 0.6 μM of chelidonine (**22**) can significantly inhibit the G2/M phase of mitosis in T98G cells [59]. Lenvatinib is capable of enhancing the apoptosis of HCC cells by chelidonine (**22**), thus inhibiting the epithelial mesenchymal transition (EMT)-related factor of HCC cells based on the possible mechanism. Moreover, chelidonine inhibits HCC cells MHCC97-H and LM-3 with IC_50_ values of 7.72 μM and 6.34 μM [60]. Chelidonine (**22**) can inhibit the cell cycle of leukemic T cells in different p53 states while suppressing tubulin polymerization in A549 cells, and the IC_50_ values of different tumor suppressor proteins’ *p53* gene for MOLT-4, HL-60, U-937, and Raji range from 4.8 to 8.3 μM [51].

**Figure 8:**
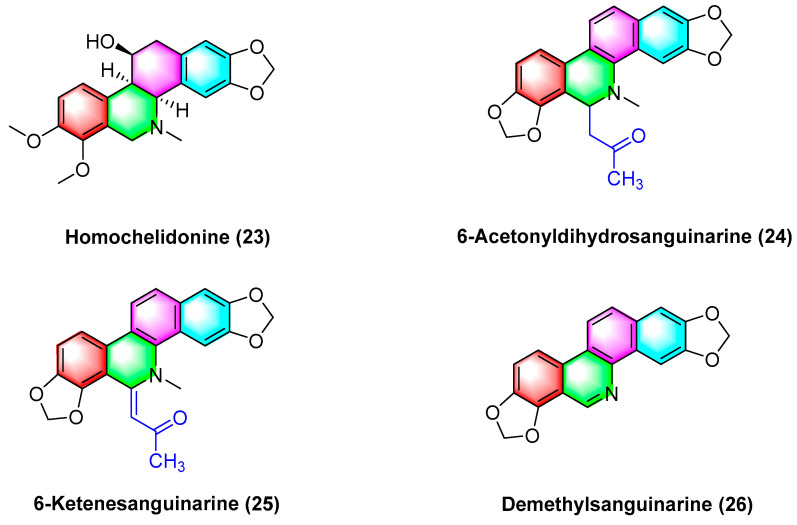
Benzophenanthridine alkaloids from *Corydalis bungeana Turcz.*

Havelek R. et al. [61] have indicated that homochelidonine (**23**) (Figure 8) induces apoptosis and arrests the G2 phase mitotic cell cycle in cancer cells, and 20 µM homochelidonine (**23**) inhibits the cell growth of SK-BR-3, HepG2, and MCF-7 by over 50%.

Acetyldihydrosanguinarine (**24**), 6-ketenesanguinarine (**25**), and demethylsanguinarine (**26**) (Figure 8) are isolated from *Corydalis bungeana Turcz*. Xiyun Ye et al. [62] determined the cytotoxic activity of the above two compounds against A549, HT-29, kb16, and P-388 cell lines, respectively, and their ED_50_ (μg/mL) values reach 1.840, 1.600, 0.340, and 0.051, respectively.

#### 2.1.4. Antitumor Activities of Benzophenanthridine Alkaloids from *Corydalis*

**Figure 9:**
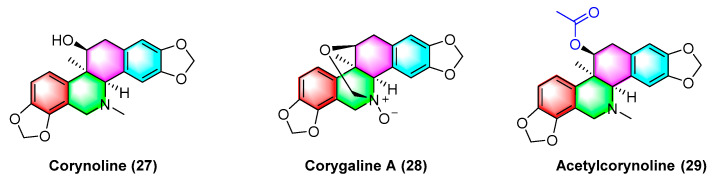
Benzophenanthridine alkaloids from *corydalis.*

Corynoline (**27**) (Figure 9) is a natural product derived from the traditional Chinese medicine *Corydalis*. It significantly inhibits the cell cycle and induces apoptosis in melanoma cells B16F10 and A375 in vivo, with an IC_50_ value of 6.16 μM. The IC_50_ value of A375 reaches 5.56 μM. The mechanism between them is correlated with the upregulated gene expression of *Bax* and cleavage of Caspase-3 [63].

Corygaline A (**28**) (Figure 9), isolated from *Corydalis bungeana Turcz*, refers to a hexahydrobenzophenanthridine alkaloid with an unusual carbon skeleton. Corygaline A (**28**) is capable of inhibiting the NO production in LPS-activated RAW264.7 macrophages with an IC_50_ value of 2.9 μM. Moreover, it is independent of dose [64].

Acetylcorynoline (**29**) (Figure 9), originating from the rhizome of the natural plant *Corydalis* incisa, inhibits the mitotic process of cancer cells by affecting chromosomes, spindles, and the cytoplasm during mitosis, which eventually arrests the mitotic process and induces apoptosis. The mitotic process of the cells is significantly inhibited by 10 μM acetylcorynoline. Acetylcorynoline (**29**) potently inhibits human colon carcinoma HCT-116, lung adenocarcinoma cell NCI-H23, lung carcinoma H460, as well as cervical carcinoma TuWi with EC_50_ values < 20 μg/mL [65].

**Figure 10:**
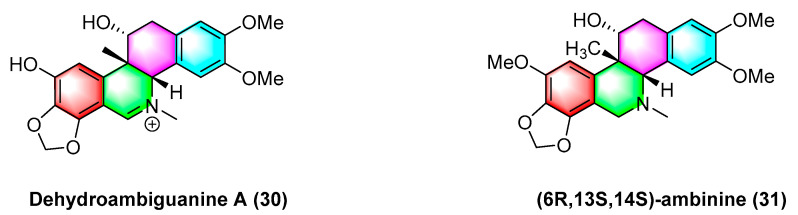
Benzophenanthridine alkaloids from *Corydalis ambigua* subsp. *Amurensis.*

As shown in Figure 10, dehydroambiguanine A (**30**) and (6*R*, 13*S*, 14*S*)-ambinine (**31**) are extracted from *Corydalis ambigua* subsp. *Amurensis*. As indicated by the result, both alkaloids can inhibit the proliferation of tumor cells during activity tests. They exhibit strong activity against the human colon cancer cell line HCT-116, dehydroambiguanine A (**30**) with an IC_50_ value of 49.8 ± 4.79 μM. Furthermore, (6*R*, 13*S*, 14*S*)-ambinine (**31**) is less active (IC_50_ values > 200 μM) [66].

#### 2.1.5. Antitumor Activities of Benzophenanthridine Alkaloids from *Macleaya cordata*

**Figure 11:**
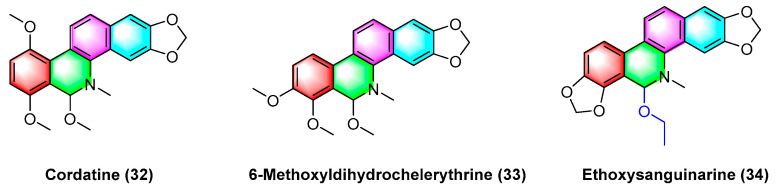
Benzophenanthridine alkaloids from the fruits of *Macleaya cordata.*

The benzophenanthridine alkaloids cordatine (**32**) and 6-methoxyldihydrochelerythrine (**33**) in Figure 11 are extracted from the fruits of *Macleaya cordata*. Hui Liang Zou et al. [67] determined the cytotoxic activity of the above two benzophenanthridine alkaloids against MCF-7 and SF-268 through the MTT assay. As revealed by the results, the IC_50_ value of cordatine (**32**) against MCF-7 cells is 34.78 mM, and that against SF-268 cells reaches 11.79 mM. Furthermore, the IC_50_ value of 6-methoxydihydrochelerythrine (**33**) reaches 21.45 mM and 4.28 mM, respectively.

Ethoxysanguinarine (**34**) is derived from *Macleaya cordata* (*Willd*) *r. br*., and ethoxysanguinarine (**34**) inhibits the anchorage-dependent and anchorage-independent growth of breast cancer cells by inducing cell autophagy by upregulating the activity of AMP-activated protein kinase (AMPK). Ethoxysanguinarine (**34**) exhibits strong activity against seven breast cancer cell lines (including MCF-7, sk-br3, MDA-MB-231, MDA-MB-436, MDA-MB-468, MDA-MB-453, and MDA-MB-435S) with IC_50_ values from 2.63 to 9.15 μM [68]. Recently, a study showed that it inhibits the viability of MCF-7 and MDA-MB-231 human breast cancer cells and induces apoptosis via a mechanism related to a Hakai-related signaling pathway [69].

**Figure 12:**
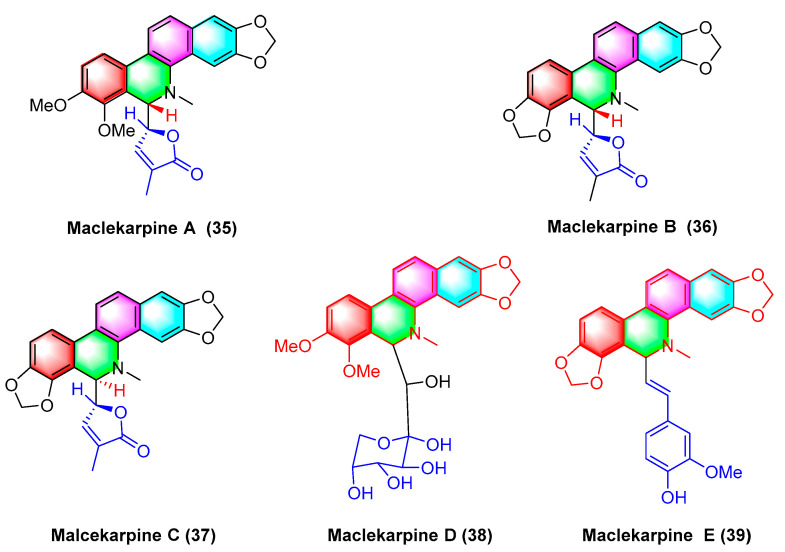
Benzophenanthridine alkaloids from the fall back roots of *Macleaya cordata.*

**Figure 13:**
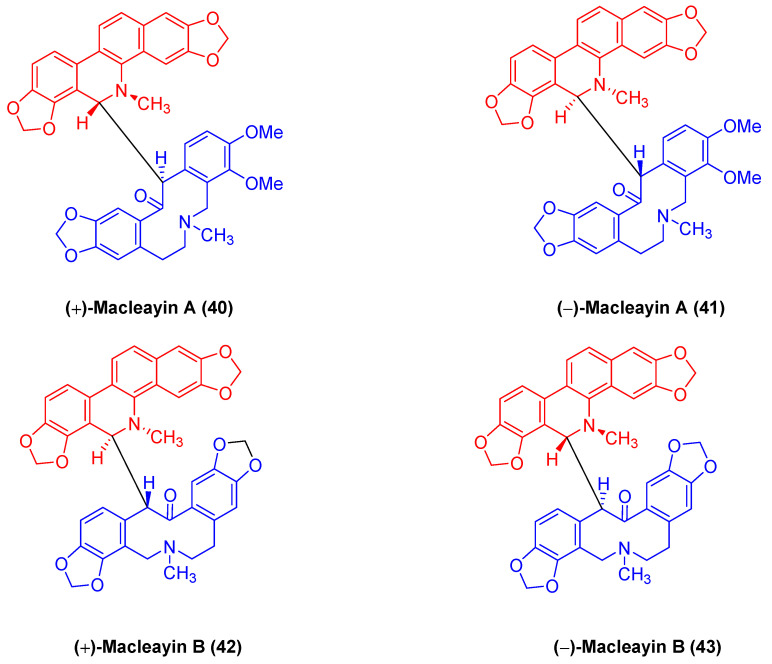
Benzophenanthridine alkaloids from *Macleaya cordata* (*Willd.*) *r. br.*

Five dihydrodibenzophenanthridine alkaloids, termed maclekarpine A–E (**35**–**39**) (Figure 12), are isolated from the fall back roots of *Macleaya cordata*. The alkaloids maclekarpine A–E (**35**–**39**) exhibit high antitumor activity and have an inhibitory effect on several human cancer cells (e.g., human colon cancer cell line HCT-8, human hepatoma cell line BEL-7402, human gastric cancer cell line BGC-823, human ovarian cancer cell line A2780, as well as human lung cancer cell line A549). Maclekarpine A (**35**) exhibits excellent activity against BGC-823 with an IC_50_ value of 0.7 μM, except that maclekarpine B (**36**) is inactive. The IC_50_ value of maclekarpine C (**37**) ranges from 1.6 to 3.4 μM. Maclekarpine D (**38**) IC_50_ values range from 0.2 to 2.0 μM. Maclekarpine E (**39**) has a significant inhibitory activity against BGC-823 with the IC_50_ value of 0.1 μM [70].

(±)-macleayin A (**40**, **41**) and (±)-macleayin B (**42**, **43**) in Figure 13 originating from *Macleaya cordata* (*Willd.*) *r. br*. are enantiomeric natural dimeric alkaloids. (±)-macleayin A (**40**, **41**) are prepared by coupling dihydrosanguinarine with allocryptamine through the 6,13′-C-C bond, and (±)-macleayin B (**42**, **43**) are synthesized by coupling dihydrosanguinarine with proto opioid through a 6,13′-C-C bond. In the in vitro activity test, (±)-macleayin A and (±)-macleayin B have significant inhibitory effects on cancer cell HL-60 with IC_50_ values from 3.51 to 9.64 μM. Macleayin A exhibits the optimal activity, while the IC_50_ values of sanguinarine and allocryptophylline reach 7.71 and 7.18 μM, respectively [71].

**Figure 14:**
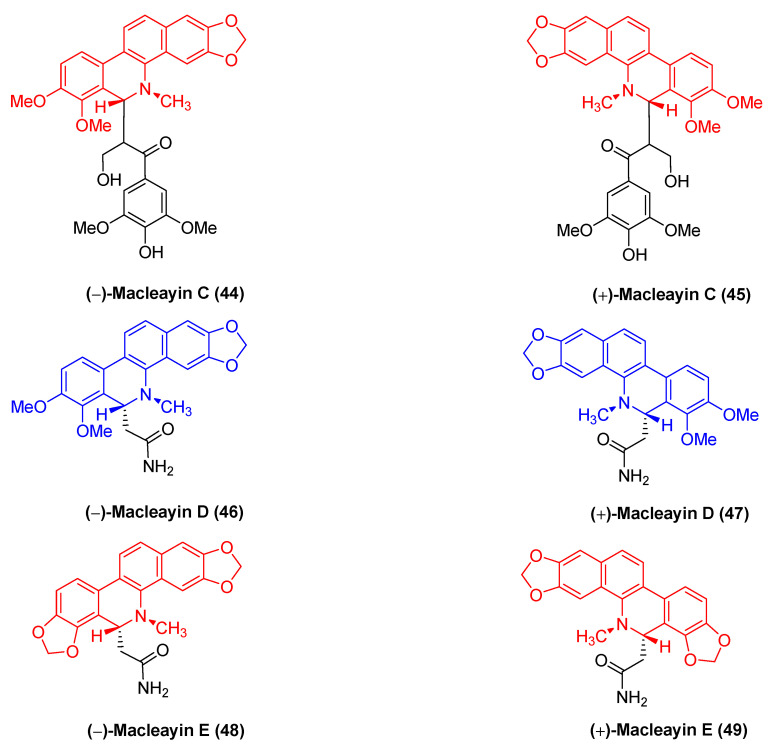
Benzophenanthridine alkaloids from *Macleaya cordata*.

The natural benzophenanthridine alkaloids (−)-macleayin C (**44**), (+)-macleayin C (**45**), (−)-macleayin D (**46**), (+)-macleayin D (**47**), (−)-macleayin E (**48**), and (+)-macleayin E (**49**) (Figure 14) with enantiomers originate from *Macleaya cordata*. They are novel compounds comprising dihydrophenanthridine alkaloids with phenylpropane. Among the cytotoxic activities, (−)-macleayin C (**44**) and (+)-macleayin C (**45**) are more active against HL-60 cancer cells with IC_50_ values of 12.13 and 10.15 μM, respectively. (−)-macleayin D (**46**), (+)-macleayin D (**47**), (−)-macleayin E (**48**), and (+)-macleayin E (**49**) significantly inhibit HL-60 and A549 cancer cells with IC_50_ values from 18.46 to 35.26 μM [72].

**Figure 15:**
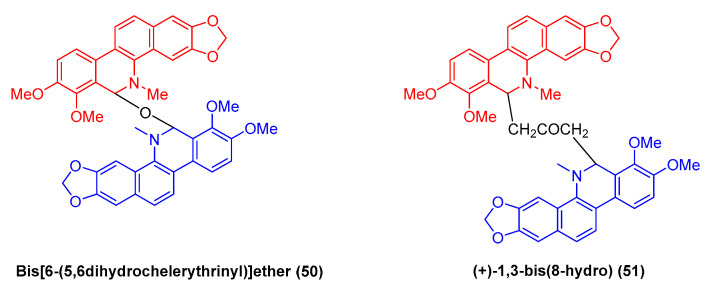
Benzophenanthridine alkaloids from *M. microcarpa*.

Bis-[6-(5,6dihydrochelerythrinyl)] ether (**50**) (Figure 15) refers to a benzophenanthridine benzophenanthridine alkaloid dimer originating from the roots of *M. microcarpa*. It exhibits favorable cytotoxic activity against human cancer cell lines (e.g., HCT-8, BEL-7402, BGC-823, and A2780) with IC_50_ values of 1.6, 2.1, 0.1, and 1.6 μM, respectively [43]. Moreover, existing research has indicated that the natural benzophenanthridine dimeric alkaloid (+)-1,3-bis (8-hydro) (**51**) exhibits antitumor activity [73].

### 2.2. Antitumor Activities of Benzophenanthridine Alkaloids from Plants of the Genus Tanneraceae

**Figure 16:**
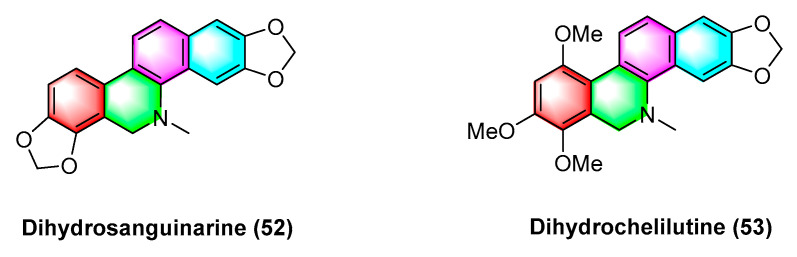
Benzophenanthridine alkaloids from *Thalictrum microgynum Lecoy ex Oliv.*

Jie Jiang et al. [74] isolated and extracted two benzophenanthridine alkaloids with anticancer activity dihydrosanguinarine (**52**) and dihydrochelilutine (**53**) (Figure 16) from *Thalictrum microgynum Lecoy ex Oliv*. Dihydrosanguinarine (**52**) inhibits pancreatic cancer cells by downregulating mut-p53 or wt-p53 protein expression and regulating the RAS/Raf/MEK/ERK pathway, and the mechanism may be correlated with inducing apoptosis in PANC-1 and SW1990 cells and triggering cell cycle arrest in PANC-1 cells [75]. Dihydrosanguinarine (**52**) at 5 μM concentration significantly inhibits human promyelocytic leukemia HL-60 cells with *p* < 0.001 while reducing cell survival [76].

### 2.3. Antitumor Activities of Benzophenanthridine Alkaloids from Rutaceae

#### 2.3.1. Antitumor Activities of Benzophenanthridine Alkaloids from *Zanthoxylum rhoifolium*

Nitidine chloride (**54**), a herb derived from the root of *Zanthoxylum avicennae*, exhibits anti-inflammatory, antifungal, anti-HIV, and antimalarial biological activities and shows high activity against tumor cancer cells [77], as indicated by the IC_50_ values (μM) against four cancer cell lines (including HepG2, A549, NCI-H460, and CNE1) that reach 1.40 ± 0.16, 1.88 ± 0.24, 2.35 ± 0.35, and 1.85 ± 0.08, respectively. Pan X et al. [78] have confirmed that nitidine chloride (**54**) can inhibit the protrusion formation and partial proteolytic activity of MMP-9 and MMP-2 in a dose-dependent manner; reduce the PDGF-induced phosphorylation of c-Src, FAK, and MAPKs; and decrease AP-1 transcriptional activity to inhibit the human breast cancer MDA-MB-231 cell line. Nitidine chloride (**54**) can inhibit HepG2, HCCLM3, and Huh7 growth, arrest G1/s cell cycle, inhibit proliferation, induce apoptosis, and suppress the expression of cegf-a and VEGFR2 in HCC cells in vitro and in vivo by activating the mitochondria-dependent pathway [79]. Huaping Mou et al. [80] have suggested that nitidine chloride (**54**) can inactivate S-phase kinase-associated protein 2 (Skp2) to inhibit ovarian cancer, mainly downregulating Skp2 expression and enhancing the sensitivity of ovarian cancer cells to nitidine chloride, in which *p* < 0.05. Nitidine chloride (**54**) is capable of inhibiting renal cancer cell 786-O and A498 cells, as confirmed by cell viability and flow cytometric apoptosis analysis. Its mechanism is primarily induced by downregulating the signaling process of p53, BcI-2, caspase-3, and inhibiting ERK [81]. Nitidine chloride (**54**) can induce autophagy and apoptosis of ovarian cancer cells through various signaling pathways, such as Akt/mTOR. It may become a potential target for ovarian cancer chemotherapy [82].

In cancer cell proliferation, nitidine chloride (**54**) can serve as a potent inhibitor while inhibiting cell growth, apoptosis, migration, and invasion in glioblastoma cell lines by downregulating the expression of calmodulin-dependent protein kinase III, which may be correlated with the activation of Cdc20 oncoprotein expression [83]. Moreover, osteosarcoma cells can be inhibited by nitidine chloride (**54**), and Hui Xu et al. [84] have indicated that nitidine chloride (**54**) inhibits the growth, migration, and invasion and induces the apoptosis of osteosarcoma cells through an MTT assay and flow cytometric analysis. The mechanism may be correlated with the inhibition of sin1 expression in osteosarcoma cells. As revealed by the result, nitidine chloride (**54**) inhibits rectal cancer HCT-116 cells by inhibiting the phosphorylation pathway of ERK, and the mechanism is the upregulation of the expression of Bax, p53, and the downregulation of the expression of caspase-3, caspase-9, and BcI-2 [85]. Hyoung Yang et al. [86] have found that nitidine chloride (**54**) inhibits human oral squamous cell carcinoma (OSCC) by inhibiting the signal transducer and activator of transcription 3 (STAT3), and the main mechanism is the downregulation of myeloid cell leukemia-1 (MCl-1) protein in HSC-3 and HSC-4 by inhibiting the STAT3 pathway. Furthermore, acute myeloid leukemia (AML) can be inhibited by nitidine chloride (**54**) by inhibiting the phosphorylation of Akt and ERK. Its main mechanism is to downregulate cyclin B1, CDK1, and BCl; upregulate p27 and Bax in AML cells; inactivate PARP; and activate caspase-3-related signaling pathways [87]. Existing research on the anticancer effect of nitidine chloride (**54**) has suggested that it is capable of inhibiting leukemia (CML) by downregulating the expression of proto oncogene c-myc, and the possible mechanism of action of nitidine chloride (**54**) is to induce apoptosis and upregulate caspase-3 and PARP-1 in K562 cells, thus enhancing the effect of imatinib on K562 cells [88]. Nitidine chloride (**54**) is also active against BRCA1-deficient cancer cells. Existing research has suggested that bicarbonate chloride (DSBs) is also active at 0.2 µM, thus significantly inhibiting MDA-436BRCA1-KO cells (*p* ≤ 0.001), and 0.4 µ m is significantly (*p* ≤ 0.001) resistant to HCC1937-BRCA1^−/−^ inhibition [89].

Some studies have indicated that bicarbonate chloride (**54**) exhibits antitumor stem cell properties (e.g., inhibiting the epithelial mesenchymal transition (EMT) and inhibiting glioma stem cell properties through the JAK2/STAT3 signaling pathway [90]). Bicarbonate chloride (**54**) also exhibited dose-dependent anti-liver cancer stem cell activity, which was confirmed in nude mice experiments [91]. Furthermore, nitidine chloride (**54**) inhibits the epithelial mesenchymal transition process while suppressing tumor stem cell properties in breast cancer cells through the hedgehog signaling pathway [92]. In the in vitro activity study, nitidine chloride (**54**) inhibited bladder cancer cells by downregulating the expression of *Lymphocyte antigen 75* (LY75) [93].

**Figure 17:**
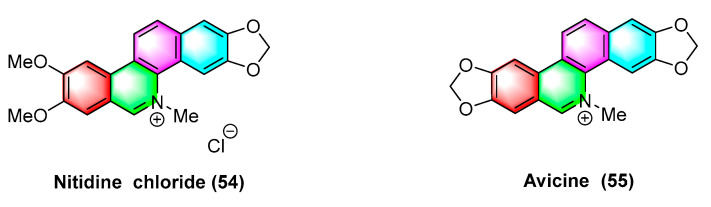
Benzophenanthridine alkaloids from *Zanthoxylum rhoifolium.*

Avicine (**55**) and nitidine chloride (**54**) (Figure 17) are two types of benzophenanthridine alkaloids derived from the roots of *Zanthoxylum avicennae* that serve as cholinesterases (AChE), monoamine oxidases A (Maos), and A β 1–42 targeted inhibitors. Erika Plaza et al. [79] analyzed the IC_50_ values for inhibiting EeAChEH, rAChE, EqAChE, MAO A, and MAO B using avicine (**55**) and nitidine chloride (**54**). The IC_50_ values (μM) of avicine-inhibited AChEW as 0.15 ± 0.01, 0.52 ± 0.05, and 0.88 ± 0.08 reach 0.41 ± 0.02, and the inhibition Maos IC_50_ values (>100 μM). However, the IC_50_ values (μM) of nitidine chloride-inhibited AChE reach 0.65 ± 0.09, 1.25 ± 0.09, and 5.73 ± 0.60, and those of inhibition MAOs IC_50_ values reach 1.89 ± 0.17 and >300 μM.

**Figure 18:**
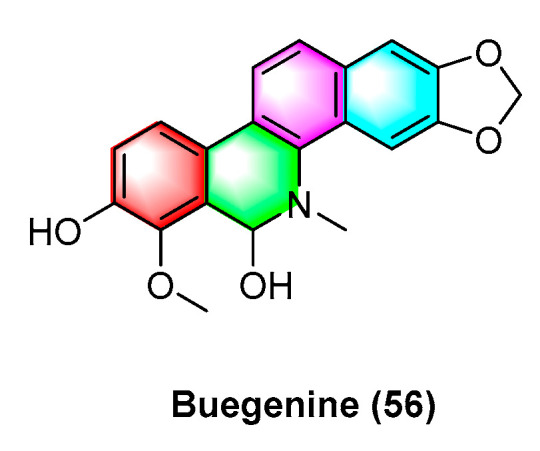
Benzophenanthridine alkaloids from *Zanthoxylum buesgenii.*

Buegenine (**56**) (Figure 18), derived from *Zanthoxylum buesgenii*, is cytotoxic in nine cancer cell lines, including leukemic cancer cells CCRF-CEM and CEM/ADR5000, breast cancer cell MDA-MB231 and its resistant subline MDA-MB231/BCRP, colon cancer cell HCT-116p53^+/+^ and its resistant subline HCT-116p53^−/−^, glioblastoma U87MG and its resistant subline U87MGΔEGFR, HepG2 hepatoma cells, and AML12 normal liver cells. It exhibits high activity with IC_50_ values less than 65 μM. Notably, buegenine (**56**) exhibits better activity against drug-resistant CEM/ADR5000 cell lines than doxorubicin [94].

**Figure 19:**
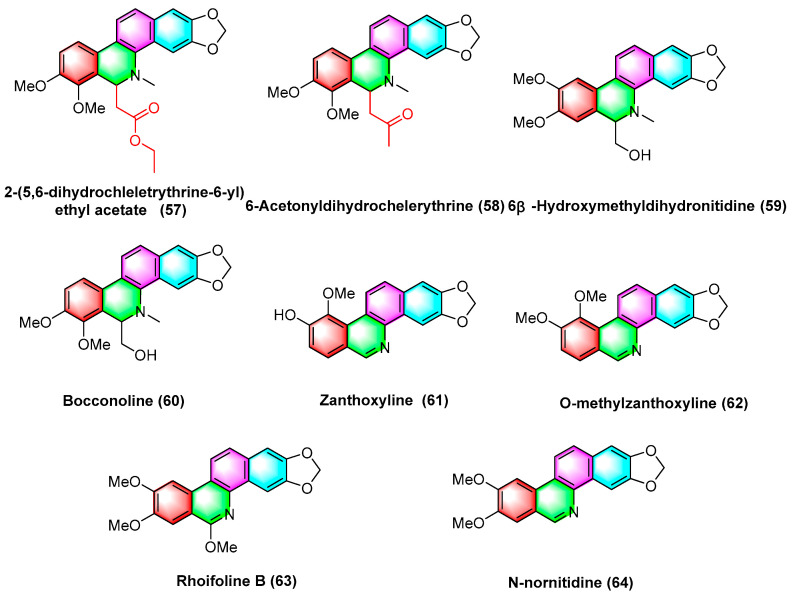
Benzophenanthridine alkaloids from *Zanthoxylum avicennae.*

A wide variety of benzophenanthridine alkaloids (Figure 19) have been extracted by Deng, Y’s group [95] in *Zanthoxylum avicennae* from Yunnan Province, and their antileukemic cell line HLa activity in vitro is determined, with better activity of bocconoline (**60**), zanthoxyline (**61**), nitidine chloride (**54**), chelerythrine (**2**), and their IC_50_ values (μM) in the order of 7.65 ± 0.11, 24.94 ± 1.99, 3.59 ± 0.82, and 15.52 ± 0.26. Nitidine chloride (**54)** results in significant S-phase arrest and induces apoptosis in HEL cells, thus becoming a promising potential antileukemic candidate. Furthermore, other alkaloids 2-(5,6-dihydrochelerythrine-6-ylethyl acetate) (**57**), 6-acetonydihy–drochelerythrine (**58**), the activities of 6 β-hydroxymethyldihydronitidine (**59**), o-methylzanthoxyline (**62**), rhoifoline B (**63**), and n-nornitidine (**64**) are less weak with IC_50_ values higher than 30 μM.

**Figure 20:**
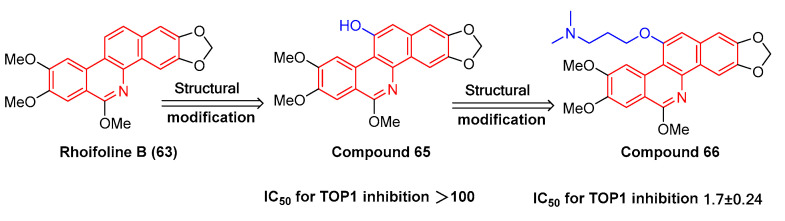
Study on structure–activity relationship of rhoifoline B.

The natural product rhoifoline B (**63**) is structurally modified to obtain compound **65** (Figure 20) with the hydroxy substitutions at the 11-position. Compound **66** (Figure 20), originating from compound **65** through structural modification, enhances the inhibition selectivity of tyrosine DNA phosphodiesterase 1 (TDP1), with an IC_50_ value of 1.7 ± 0.24 μM. It is also capable of inhibiting the enhancement of DNA topoisomerase IB (TOP1) activity, thus effectively inhibiting MCF7, A549, H460, and HepG2 cells. The structure–activity relationship study indicates that the 11 hydroxyl groups of compound **65** are replaced by different chemical structures. For instance, compounds with triazole branched chains exhibit poor activity, which may be correlated with poor cell permeability [96].

**Figure 21:**
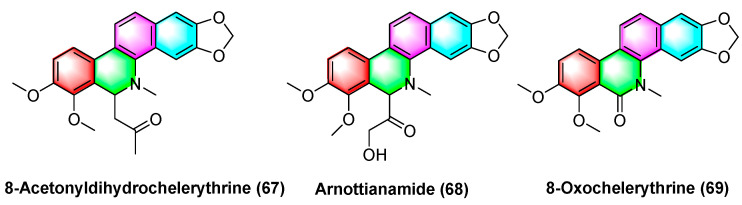
Benzophenanthridine alkaloids from *Zanthoxylum paracanthum Kokwaro.*

In Figure 21, the benzophenanthridine alkaloids 8-acetyldihydrocheleryrhtine (**67**), arnottianamide (**68**), and 8-oxochelerythrine (**69**) with anticancer effects are extracted and isolated from *Zanthoxylum paracanthum kokwaro*. Several studies have found that 8-acetyldihydrocheleryrhtine (**67**), arnottianamide (**68**), and 8-oxochelerythrine (**69**) can inhibit human breast cancer cell line HCC-1395 and human prostate cancer cell DU 145. The IC_50_ value for 8-acetyldihydrochelerythrine (**67**) reaches 9.99 μg/mL and 66.82 μg/mL. To be specific, the IC_50_ value of zanthoxylamide (**68**) reaches 38.34 μg/mL and 84.31 μg/mL. The IC_50_ value of 8-oxovalerythrine (**69**) reaches 14.09 μg/mL and 63.41 μg/mL [97].

**Figure 22:**
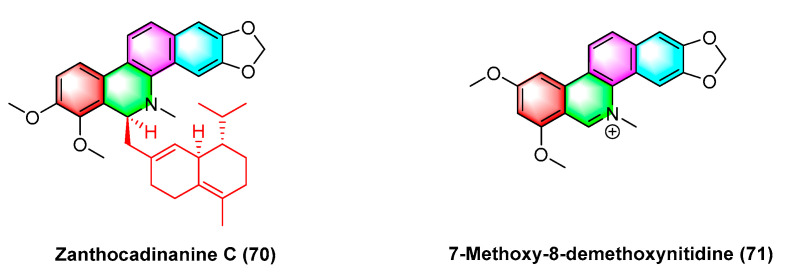
Benzophenanthridine alkaloids from *Zanthoxylum nitidum* (*Roxb.*) *DC.*

Two benzophenanthridine alkaloids with cytotoxic activity against a variety of human cancer cell strains, zanthocadinanine C (**70**) and 7-methoxy-8-demethoxynitidine (**71**) (Figure 22), are isolated from the traditional drug *Zanthoxylum nitidum* (*Roxb.*) *DC*. Zanthocadinanine C (**70**) and 7-methoxy-8-demethoxynitidine (**71**) exhibit cytotoxic activity against five human cancer cell lines KB, MCF-7, LNCaP, HepG-2, and lu-1, whereas the alkaloid 7-methoxy-8-demethoxynitidine (**71**) has high activity with IC_50_ values from 10.3 to 12.6 μM. Furthermore, zanthocadinanine C (**70**) is less active with IC_50_ > 100 μM [98].

**Figure 23:**
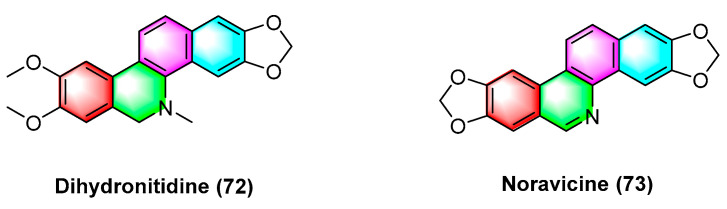
Benzophenanthridine alkaloids from *Zanthoxylum.*

Dihydronitidine (**72**) (Figure 23), a benzophenanthridine alkaloid, is isolated from *Zanthoxylum*. Dihydronitidine exhibits high antitumor activity, and its mechanism may be the regulation of cell cycle-related genes *CDK2* and *CCNE* in tumor cells and the upregulation of the expression of relevant genes of apoptosis. For six different cancer cell lines, including lung cancer cell A549, colon adenocarcinoma cell COLO-201, pancreatic cancer cell MIA-PaCa2, epidermoid carcinoma cell A431, gastric cancer cell KATO III, and breast cancer cell SKBR-3, the ED_50_ values of dihydrobifrontal bases range from 0.19 to 4.60 μg/mL [99].

Noravicine (**73**) (Figure 23), a benzophenanthridine alkaloid isolated from the roots of nitidum needles of Zanthoxylum avicennae, exhibits strong cytotoxicity against Ball-1 cancer cells with IC_50_ values of 74.8 ± 5.93 μM [89].

**Figure 24:**
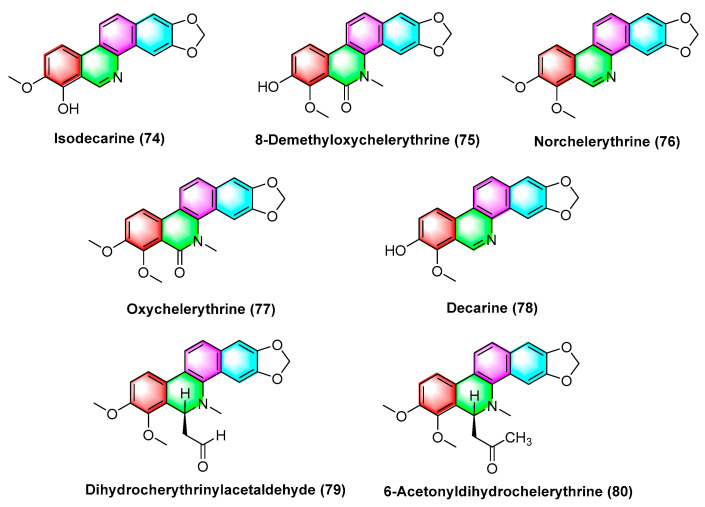
Benzophenanthridine alkaloids from *Zanthoxylum integrifoliolum.*

The isolated benzophenanthridine alkaloids extracted from *Zanthoxylum integrifoliolum* are isodecarine (**74**), 8-Demethyloxychelerythrine (**75**), Norchelerythrine (**76**), Oxychelerythrine (**77**), Decarine (**78**), Dihydrocherythrinylacetaldehyde (**79**), and 6-Acetonyldihydrochelerythrine (**80**) (Figure 24). Compounds **74**–**80** exhibit potent toxicity against P-388 and HT-29 cancer cell lines with ED_50_ values from 1.15 to 10.30 μg/mL, where dihydrocherythrinylacetaldehyde (**79**) exhibits the optimal activity, with ED_50_ values of 1.15 and 4.08 μg/mL, respectively [100].

**Figure 25:**
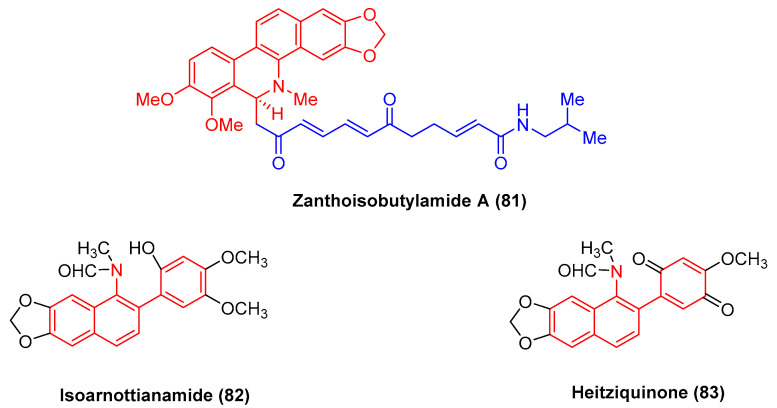
Benzophenanthridine alkaloids from *Zanthoxylum nitidum.*

The alkaloid zanthoisobutylamide A (**81**) (Figure 25), isolated from Zanthoxylum nitidum roots by extraction, refers to a dihydrobenzophenanthridine alkaloid dimer linked with an unsaturated alkylamide through a C-6 bond. Zanthoisobutylamide A (**81**) potently inhibits human epidermal carcinoma A431 cells with the IC_50_ value of 29.75 μg/mL [101].

Isoarnottianamide (**82**) and heitziquinone (**83**) (Figure 25) refer to a new class of benzophenanthridine alkaloids. To be specific, heitziquinone (**83**) was isolated from the subfractions of isoarnottianamide (**82**), and its toxic activity against RAW 264.7 cells was examined [102].

#### 2.3.2. Antitumor Activities of Benzophenanthridine Alkaloids from Fagaropsis

**Figure 26:**
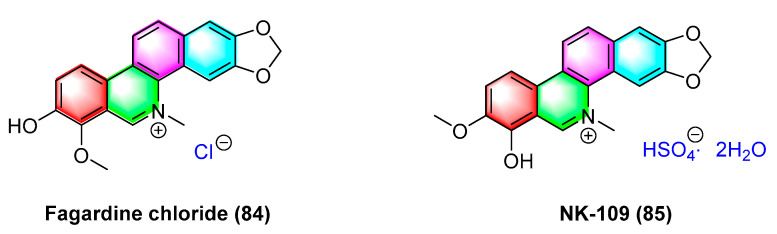
Benzophenanthridine alkaloids from *Fagara tessmannii.*

Fagaridine chloride (**84**) (Figure 26), a benzophenanthridinium natural product isolated from *Fagara tessmannii*, has shown promising antitumor effects, and the mechanism is correlated with the activation of caspases, the alteration of MMPs, and the secretion amount of ROS. Fagaridine chloride (**84**) exhibits potent cytotoxicity against several cancer cell lines (e.g., CCRF-CEM cells, CEM/ADR5000 cells, MDA-MB-231-pcDNA3 cells, MDA-MB-231-BCRP clone 23 cells, HCT-116p53 ^+/+^, HCT-116p53^−/−^ cells, U87.MG cells and counterparts U87.MGΔEGFR, and HepG2 cells). The IC_50_ values range from 1.69 to 63.38 μM. Furthermore, it exhibits strong cytotoxicity against CCRF-CEM cells, CEM/ADR5000, and U87.MG cells [103].

NK-109 (**85**) (Figure 26) refers to a benzophenanthridinium salt alkaloid that is structurally similar to fagaridine but contains a phenolic hydroxyl substitution at C-7. It is a topoisomerase II inhibitor with better activity. Its metabolites are less toxic to host cells and exhibit significant antitumor activity in clinical trials [104], and NK-109 (**85**) exhibits significant inhibitory activity against a mixture of HeLa S3 cells and human liver S-9 with IC_50_ values of 0.08 μg/mL [105] and 0.32 μM [104]. The IC_50_ values for K562/ADM cells, adrr MCF7 cells, PC-9/CDDP cells, and SKOV3/VP cells are obtained as 0.045, 0.42, 0.19, and 0.21 μg/mL, respectively [106]. Research has shown that 6-substituted derivatives of NK-109 will affect their antitumor activity. The 8-*O*-substituted derivatives partially inhibit biological reduction. Bulky hydrophobic substituents will weaken activity, while hydrophilic substituents show similar activity as NK-109. The 5-substituted derivatives show strong activity [104].

**Figure 27:**
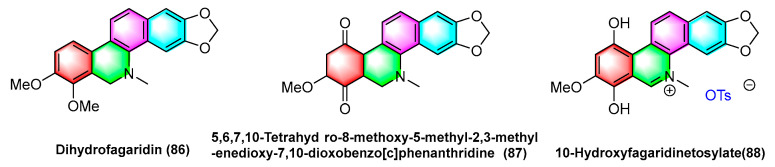
Benzophenanthridine alkaloids from *fagaropsis.*

The natural product fagaridine chloride (**84**) is structurally modified to compounds dihydrofagaridin (**86**), 5,6,7,10-tetrahydro-8-methoxy-5-methyl-2,3-methyl-enedioxy-7,10-dioxobenzophenanthridine (**87**), and 10- hydroxyfagaridinetosylate (**88**) (Figure 27). The compounds dihydrofagaridin (**86**), 5,6,7,10-Tetrahyd ro-8-methoxy-5-methyl-2,3-meth- yl-enedioxy-7,10-dioxobenzo[*c*]phenanthridine (**87**), and 10-hydroxyfagaridinetosylate (**88**) significantly inhibit non-small cell lung cancer cell line A-549, ovarian cancer cell line SKOV-3, colon cancer cell line HCT-15, colon cancer cell line XF-498, and melanoma cell line SK-Mel-2 [107].

#### 2.3.3. Antitumor Activities of Benzopheridine Alkaloid from *Toddalia*

Benzophenanthridine alkaloid, 8-acetonyldihydronitidine (**89**), extracted from *Toddalia asiatica*, can induce p53 expression, enhance caspase-3 activity, and exhibits good antitumor activity. It is capable of significantly inhibiting colorectal cancer HCT-116 and tumor cell proliferation and inducing apoptosis in vivo, with an IC_50_ value of 12.91 μM [108].

**Figure 28:**
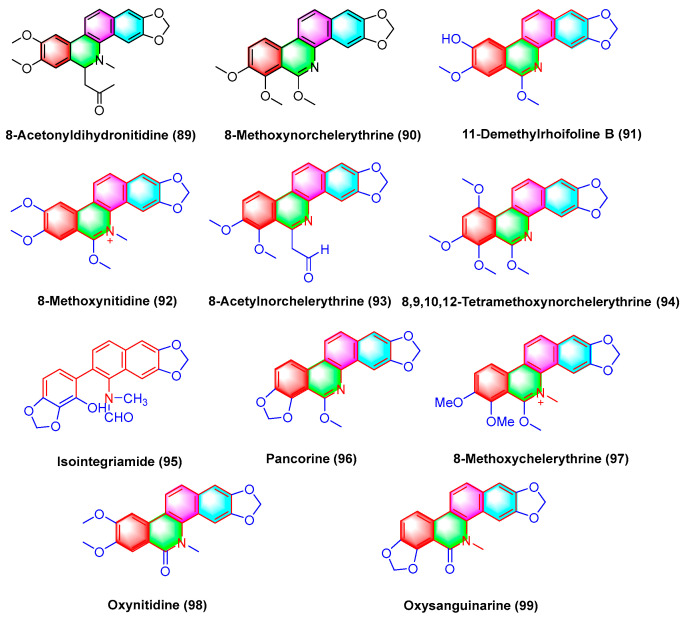
Benzophenanthridine alkaloids from *Toddalia asiatica.*

Benzophenanthridine alkaloids 8-methoxynorchelerythrine (**90**), 11-demethylrhoifoline B (**91**), 8-methoxynitidine (**92**), 8-acetylnorchelerythrine (**93**), 8,9,10,12-tetramethoxynorchelerythrine (**94**), isointegriamide (**95**), pancorine (**96**), 8-methoxychelerythrine (**97**), oxynitidine (**98**), and oxysanguinarine (**99**) (Figure 28) are extracted from *Toddalia asiatica*. Studies have shown that the above compounds could inhibit eight types of human tumor cell lines in vitro, including A549 (human lung cancer), BGC-823 (human gastric cancer), HCT-15 (human colon cancer), Hela (human cervical cancer), HepG2 (human liver cancer), MCF-7 (human breast cancer), SK-MEL-2 (human skin cancer), and SGC-7901 (human gastric cancer). Their IC_50_ values range from 1.3 to 17.8 μg/mL [109].

**Figure 29:**
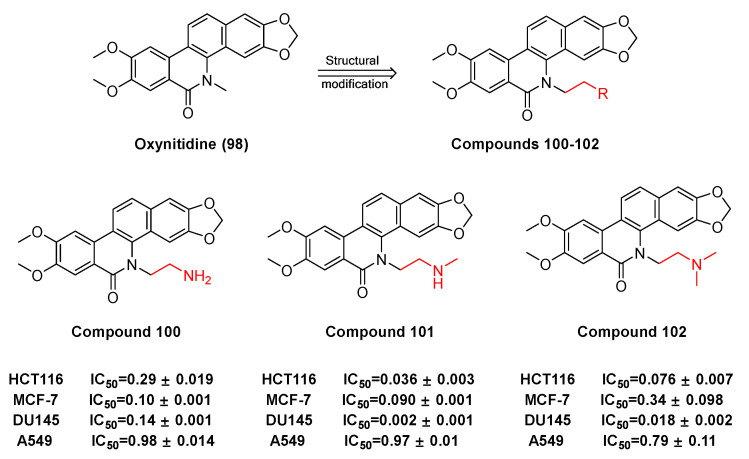
Study on structure–activity relationship of oxynitidine.

Some derivatives (**100**, **101**, **102**) (Figure 29) originate from the benzophenanthridine natural product oxynitidine (**98**) after semi synthetic structure modification. W.-L. Tang et al. [110] had suggested that the derivatives of oxynidine are capable of inhibiting the growth and proliferation of human cancer cells and inducing apoptosis by inhibiting the activity of DNA topoisomerase IB (TOP1). For HCT-116, MCF-7, DU145, and A549 cancer cell lines, the GI_50_ ± SD of camptothecin (CPT) (μM) reaches 0.009, 0.012, 0.21, and 0.099, respectively. As the derivatives of topoisomerase IB inhibitors **100**, **101,** and **102**, they inhibit the GI_50_ ± SD of HCT-116, and MCF-7, DU145, and A549 (μM), respectively, reach 0.29, 0.10, 0.014, and 0.98; 0.036, 0.090, 0.002, and 0.97; 0.076, 0.34, 0.018, and 0.79. Compared with camptothecin, compounds **100**, **101,** and **102** have better efficacy in the treatment for DU145 cancer cells.

## 3. Conclusions and Perspectives

The natural benzophenanthridine alkaloids have aroused increasing attention for their extensive and significant biological activities and favorable therapeutic effects in many aspects (e.g., anti-inflammatory, antitumor, antibacterial, and antifungal). Natural benzophenanthridine alkaloids (e.g., sanguinarine, chelerythrine, and nitidine) have achieved a high potential application value and bright application prospect. Studies have shown the potential therapeutic effects of 90 natural benzophenanthridine alkaloids on tumor cells, including human leukemia (e.g., HL-60, THP-1, MT-4, CEM, and U937), human prostate cancer (e.g., DU-145, LNCaP, and PC3), human epidermoid cancer (e.g., A431 and nheks), human pancreatic cancer (e.g., AsPC-1 and BXPC-3), human melanoma (e.g., M4Beu, A372, and OCM-1), human non-small cell lung cancer (A549), human breast cancer (e.g., MCF-7 and MDA-MB-231), human ovarian adenocarcinoma (OVCAR-3), cervical cancer (hen-16-2, HeLa), human colon cancer (HCT-116, SW480), human gastric cancer cell (BGC-823), etc. Moreover, the anticancer activity of various natural benzophenanthridine alkaloids has been verified in animal anticancer experiments. For instance, sanguinarine (**1**) exhibits antitumor properties against human and rodent breast cancer cells in vitro and in vivo. Nitidine chloride reduced the tumor volume and weight by inhibiting ERK, STAT3, and SHH pathways and regulating Bcl-2, Bax, CDK1, and VEGF pathways in mouse experiments [96]. Moreover, the activity of a few natural benzophenanthridine alkaloids is better than that of marketed drugs. For instance, burgenine (**56**) is more active than Doxorubicin in drug-resistant CEM/ADR5000 cell lines. Oxynitidine derivatives **100**, **101**, and **102** exhibit better inhibitory activity on DU145 cells than camptothecin (CPT). Existing research has suggested that natural benzophenanthridine alkaloids exhibit extensive anticancer activity.

Natural benzophenanthridine alkaloids have promising applications in the fight against the drug resistance of tumors. For instance, NK-109 (**85**) exhibits strong cytotoxicity to drug-resistant cell lines K562/ADM, AdrR MCF7, PC-9/CDDP, and SKOV3/V, with IC_50_ values of 0.045, 0.42, 0.19, and 0.21 μg/mL, respectively, and the drug molecule has entered the clinical research stage. In addition, the natural products of benzophenanthridine exhibit significant cell activity against cancer stem cells. Sanguinarine (**1**) exerts a specific targeting effect on lung cancer stem cells, and it is a natural drug-resistant compound against lung cancer. In addition, sanguinarine (**1**) inhibits pancreatic cancer stem cells by inhibiting the sonic hedgehog signaling pathway. Chelerythrine (**2**) downregulates β- Catenin, thus inhibiting non-small cell lung cancer stem cells. Nitidine chloride (**54**) inhibits epithelial mesenchymal transformation (EMT) and glioma stem cell characteristics by the JAK2/STAT3 signaling pathway. Furthermore, it exhibits a dose-dependent antihepatoma stem cell activity, as confirmed in nude mouse experiments. Moreover, it can inhibit the epithelial mesenchymal transformation of breast cancer cells and suppress the characteristics of tumor stem cells through the hedgehog signaling pathway. Ferroptosis takes on critical significance in the treatment of cancer stem cells [111]. Recent research has revealed that sanguinarine (**1**) can induce H_2_O_2_-dependent cell ferroptosis in human cervical cancer (HeLa) by downregulating SLC7A11 and GSH [6].

## Figures and Tables

**Table 1:** The IC_50_ value of compounds **8**–**12**.

Compound	R	IC_50_ Value	
		Jurkat Clone e6-1	THP-1
Sanguinarine	/	1.56 ± 0.09	1.60 ± 0.13
Compound **8**	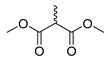	1.30 ± 0.05	1.46 ± 0.06
Compound **9**	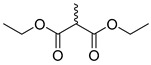	0.52 ± 0.03	0.48 ± 0.03
Compound **10**	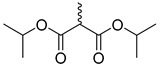	1.23 ± 0.08	1.38 ± 0.04
Compound **11**	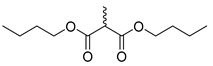	0.91 ± 0.04	1.17 ± 0.13
Compound **12**	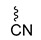	0.53 ± 0.05	0.18 ± 0.03

**Table 2:** The IC_50_ values of compounds **13**–**16**.

Compound	R	IC_50_ Value	
		Jurkat Clone e6-1	THP-1
Chelerythrine	/	5.58 ± 0.13	4.70 ± 0.07
Compound **13**	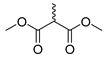	7.94 ± 0.10	5.78 ± 0.23
Compound **14**	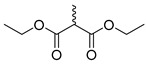	2.61 ± 0.19	1.87 ± 0.22
Compound **15**	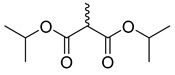	2.48 ± 0.13	4.45 ± 0.34
Compound **16**	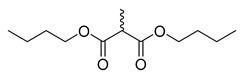	5.64 ± 0.20	5.88 ± 0.07

## Data Availability

Not applicable.
